# Probing the Rydbergization of Water through the Stabilization Method

**DOI:** 10.1021/acsphyschemau.5c00058

**Published:** 2025-08-13

**Authors:** Pedro A. S. Randi, Paulo Limão-Vieira, Márcio H. F. Bettega

**Affiliations:** † Departamento de Física, 28122 Universidade Federal do Paraná, Postal Code 19044, 81531-980 Curitiba, Paraná, Brazil; ‡ Atomic and Molecular Collisions Laboratory, CEFITEC, Department of Physics, NOVA School of Science and Technology, 50106 Univerisdade NOVA de Lisboa, Postal Code 2829-516 Caparica, Portugal

**Keywords:** water, rydbergization, stabilization method, rydberg-to-valence conversion, electronically excited states

## Abstract

A molecular orbital or electronically excited state may change its character, from Rydberg or mixed valence-Rydberg to valence, as dissociation progresses. This geometrical dependency of the electronically excited states is known as Rydbergization. Recently, we proposed a new approach to characterizing the nature of electronically excited states based on the stabilization method [ RandiP. A. S.,  J. Phys. Chem. A 2025, 129, 5820–5828 40555669 10.1021/acs.jpca.5c02982PMC12235643]. Here, we demonstrate that the stabilization method can effectively describe the Rydbergization phenomenon in the low-lying excited states of water. To this end, we analyze both the symmetric and asymmetric dissociation pathways, comparing our findings to previously reported results whenever possible. In addition to reproducing established data, we present new insights into the symmetric dissociation of states with *B*_2_ symmetry, as well as previously unexplored behavior along the asymmetric dissociation pathway. We conclude also that Rydbergization is pathway-dependent and that conclusions drawn from one geometric distortion cannot be uncritically generalized to others.

## Graphical abstract

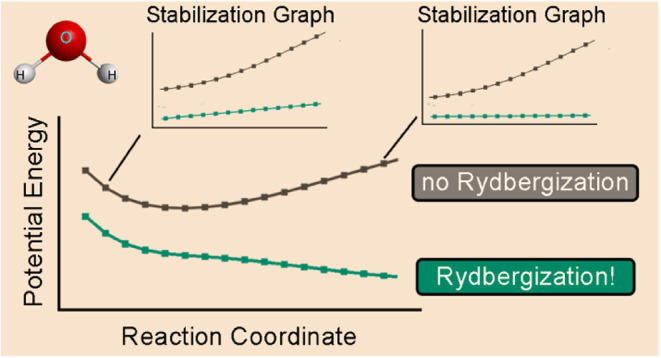

## Introduction

When molecules interact with fundamental particles such as photons, electrons, or positrons, electronic excitation can occur. The electronically excited states accessed through these interactions play a crucial role in the chemistry of a wide range of environments.^1–8^ These excited states may exhibit valence, Rydberg, or mixed valence-Rydberg character. As classified by Mulliken,^9^ valence states are associated with transitions involving well-localized molecular orbitals (MOs) with bonding or antibonding character between atoms, such that the spatial extent of the occupied MOs in a valence electronically excited state is comparable to that of the occupied MOs in the electronic ground state. On the other hand, Rydberg states involve transitions to diffuse MOs, where the MOs occupied by the excited electron are much larger than the ones occupied in the electronic ground state and have a considerable resemblance to atomic orbitals whose energies are close to the ionization energy. Mixed valence-Rydberg states are in between the former two, and can result either from configuration mixing between transitions to different MOs or from a single transition to a MO with inherently mixed character. Mixed valence-Rydberg states are also referred to as semi-Rydberg or near-Rydberg states.^9^ Understanding the nature of electronically excited states is valuable from multiple perspectives. For example, valence excitations typically lead to broad features in photoabsorption cross sections, while transitions to Rydberg states produce sharp, well-defined peaks.^10,11^ Furthermore, excited states of different character respond distinctly to their surrounding environment: valence states are typically less affected by the medium, whereas Rydberg states often undergo blue shifts due to stronger interactions with the environment.^12,13^

The character of an electronically excited state may evolve as the molecular geometry relaxes following excitation. In particular, an initial Rydberg state can transform into a valence nonbonding state during dissociation—a phenomenon known as Rydbergization.^9,14^ This transformation can occur via two distinct mechanisms. In the so-called MO Rydbergization, an unoccupied MO changes its character directly as the nuclear geometry changes. Consequently, if an electronically excited state is predominantly described by a transition to this MO, its nature also changes along the dissociation pathway. Alternatively, the character of an excited state can change adiabatically due to avoided crossings in the potential energy curves (PECs) with other states of different nature as the nuclei relax. These cases are referred to as MO-or-state Rydbergization. It is important to emphasize that in the case of MO Rydbergization, the change in character arises solely from the transformation of the MO itself, with no interaction between neighboring electronic states.^9,14^

These Rydbergization processes have been observed for many molecules.^10,11,15–21^ Especially, the Rydbergization of the low-lying electronically excited states of water has been investigated experimentally^12,21^ and theoretically.^22,23^ Chergui and Schwentner^21^ found evidence of the MO Rydbergization of the first electronically excited singlet state of water, 1^1^ *B*_1_, using matrix-isolation spectroscopy. The 1^1^ *B*_1_ state of water gives rise to a broad photoabsorption band between 6.5 and 8.5 eV,^24^ and is known to dissociate into OH + H fragments, with OH in the ground state *X̃* ^2^Π.^25,26^ Dutuit et al.^12^ studied the photodissociation of water and discussed qualitative features of the measured bands in terms of Rydbergization of states belonging to the *nsa*_1_ series. Horsley and Fink^22^ studied some excited states of water through an early self-consistent field (SCF) calculation, finding evidence of Rydbergization for the first excited singlet and triplet states of *A*_1_ symmetry. The first electronically excited states of this symmetry, 2^1^ *A*_1_, is responsible to produce a broad band in the photoabsorption spectrum between 8.7 and 9.9 eV.^24^ Rubio et al.^23^ conducted a thorough analysis of the excited states of water using MS-CASPT2 calculations. They found that, at the equilibrium geometry of the ground state, the excited states predominantly exhibit Rydberg character. However, by computing PECs for the low-lying *B*_1_, *A*_2_, and *A*_1_ singlet states along a symmetric dissociation coordinate, they showed that three states undergo Rydbergization, acquiring valence character at extended bond lengths.

The evaluation of the Rydbergization process in the theoretical calculations mentioned were based either on the analysis of the occupied orbitals as the bond stretched,^22^ or on values of the electronic radial spacial distribution.^23^ Recently, we have proposed a new approach to characterize the nature of electronically excited states in molecules, based on the stabilization method (SM).^27^ In this strategy, a series of calculations are performed in which the diffuseness of the basis set is systematically varied. Valence excited states are unaffected by these changes, exhibiting stable excitation energies. In contrast, Rydberg states show a pronounced sensitivity to the extent of the basis set.

Thus, a natural question arises: Is the SM capable of capturing the Rydbergization process? In this work, we aim to address this question using the low-lying singlet states of water as test cases. As a first step, we replicate the findings of Rubio et al.^23^ by following the symmetric dissociation pathway, in which both O–H bonds are stretched simultaneously. In addition, we perform new calculations for singlet states of *B*_2_ symmetry following this dissociation path. These calculations for the *B*_2_ symmetry increase the information regarding the Rydbergization of the low-lying electronically excited states of water, since this phenomenon is state-dependent. It is well established that the first absorption bands of water lead to dissociation into OH and H fragments.^25,26^ This process proceeds along the asymmetric dissociation pathway, rather than the symmetric one. Theoretically, Rydbergization along this asymmetric coordinate has only been explored in the early SCF study by Horsley and Fink for states belonging to the second absorption band.^22^ Thus, a second question arises: How state-of-the-art quantum chemical methods capture the Rydbergization phenomenon of the low-lying singlet states of water along the asymmetric dissociation pathway? After demonstrating that the stabilization method can accurately describe the Rydbergization process by comparing our symmetric stretching results with those of Rubio et al.,^23^ we extend our analysis to tackle this second question. As a result, in addition to proving that the stabilization method is capable of addressing Rydbergization and providing new data for the low-lying electronically excited states of water, we conclude that the Rydbergization process is pathway-dependent, and one should not infer the behavior of an electronically excited state prior to probing it adequately.

The remaining of this paper is organized as follows: in the next section, the SM approach for neutral electronically excited states is discussed, alongside the chosen underlying electronic structure method. Afterward, results for the equilibrium geometry, symmetric and asymmetric dissociation are shown and discussed. Finally, a brief conclusion is drawn from the results presented here.

## Methods

The SM was originally proposed by Hazi and Taylor to study resonances in scattering problems.^28^ Resonances are discrete states embedded within the continuum of scattering states. One instance where these states play a major role is in low-energy electron-molecule interactions. In this context, low-energy electrons can be captured by molecules present in a gas or plasma, forming a transient negative ion. This ionic state is embedded in the continuum of states associated with a free electron and a neutral molecule, thereby yielding a resonant state. Such states can lead to molecular dissociation, influencing the species present in the environment. Therefore, it is important to have reliable methods for studying these resonant states. When such scattering problems are treated using a finite basis set, as is usually the case for bound-state calculations, the continuum is not properly represented. This leads to the appearance of both true resonant states and states arising from the discretization of the continuum (DC). Thus, the assignment of resonant states is not as direct as one might hope, and one may resort to the stabilization method to make such a distinction. The procedure involves performing a series of calculations in which a parameter of the calculation is systematically varied. In the case of electron-molecule resonances, the diffuseness of the basis set is usually taken as the stabilization parameter.^29^ While true resonances remain relatively stable under these changes, DC states exhibit significant fluctuations in energy.

Recently, we extended the applicability of the SM to characterize the nature of electronically excited states in neutral molecules, applying it to CCl_4_ (carbon tetrachloride), HCOOH (formic acid), and C_7_H_7_Cl (2-chlorotoluene).^27^ The key insight is that Rydberg-like states are highly sensitive to the diffuseness of the basis set, while valence states remain stable due to their localized nature. Thus, by systematically varying the diffuseness of the basis functions, we can probe the character of an excited state. More specifically, as the basis set becomes more compact, the vertical excitation energy of Rydberg states varies drastically. In contrast, valence states, being spatially localized, exhibit minimal sensitivity to such changes and the computed vertical excitation energy remains unchanged. Additionally, mixed state with a strong valence component have a low slope in the stabilization plots, whereas mixed state with a strong Rydberg component cannot be distinguished from purely Rydberg states. Therefore, by monitoring the vertical excitation energy as a function of a stabilization parameter that controls the diffuseness of the basis set, one can effectively distinguish between valence and Rydberg or mixed-character excited states. This stabilization parameter, denoted by α, is a scaling factor to the exponents of the most diffuse functions in the basis set. Since most quantum chemical calculations use Cartesian Gaussian functions as a basis set, by increasing α, the diffuse functions become more compact.

Traditional approaches for characterizing electronically excited states, such as MO analysis or the evaluation of the electronic radial spatial distribution, often involve a degree of subjectivity.^9,27^ In MO’s analysis, one plots the occupied MOs by the excited electron in the dominant configuration of a given electronically excited state and analyses its spatial extent. However, there is no strict criterion for determining how diffuse or compact an orbital must be to classify it as Rydberg or valence. Similarly, when using the electronic radial spatial distribution, there is no rigorous threshold for deciding how closely an excited state’s distribution must match that of the ground state to be considered valence in character. In contrast, such subjectivities are absent in the SM,^27^ which provides a more systematic and objective framework for distinguishing valence state from Rydberg and mixed valence-Rydberg excited states, since it does not rely on the character of the MOs involved in the dominant configurations.

To apply the stabilization procedure, one must first choose an underlying electronic structure method to compute the electronically excited states’ vertical excitation energies. Fortunately, a plethora of high-level electronic structure calculations have already been reported for water in the literature. In this work, we adopt the EOM-CCSD/d-aug-cc-pVTZ level of theory,^30–34^ which offers a good balance between computational cost and accuracy. Comparisons with results obtained using other methods are shown in Table 1, and will be discussed in the following section. Calculations were carried out at the experimental equilibrium geometry of water,^35^ with the molecule lying in the *yz* plane. The *z-*axis was chosen to coincide with the molecular *C*_2_ symmetry axis. Geometries along the dissociation pathways were generated by modifying this reference geometry accordingly. All calculations were performed with Psi_4_.^36^

**Table 1:** Vertical Excitation Energies (in eV) for the First 4 Singlet Excited Electronic States of Water of Each Symmetry^a^

	EOM-CCSD	MS-CASPT2 ^23^	TD-DFT/HTCH(AC)^37^	GMS-SU-CCSD^38^	exFCI^39^	
state	d-aug-cc-pVTZ	ANO-L-VII	d-aug-cc-pVTZ	aug-cc-pVTZ	aug-cc-pVQZ	expt.
2^1^ *A*_1_	9.870	9.86	9.76	9.91	10.02	9.671,^24^ 9.7 ^40,41^
3^1^ *A*_1_	10.217	10.15	10.23	11.32		10.163,^24^ 10.16,^40^ 10.17 ^41^
4^1^ *A*_1_	11.470	11.09		13.31		11.12,^41^ 11.13 ^42^
5^1^ *A*_1_	12.297	12.21		13.68		
1^1^ *A*_2_	9.354	9.27	9.36	9.33	9.46	9.1 ^40^
2^1^ *A*_2_	10.893	10.84	10.85	11.65		10.84 ^40^
3^1^ *A*_2_	11.281			12.67		
4^1^ *A*_2_	12.098			15.76		
1^1^ *B*_1_	7.600	7.50	7.61	7.57	7.68	7.447,^24^ 7.4 ^40,41^
2^1^ *B*_1_	10.000	9.95	10.00	10.78		10.011,^24^ 10.01,^40^ 9.99 ^41^
3^1^ *B*_1_	10.622	11.03	10.65	11.44		10.52,^40^ 10.99 ^42^
4^1^ *B*_1_	11.101	11.19		12.89		
1^1^ *B*_2_	11.184	11.05	11.54	11.67		11.05 ^42^
2^1^ *B*_2_	11.691	11.69		13.47		
3^1^ *B*_2_	13.173			13.86		
4^1^ *B*_2_	13.493			14.20		

a The present calculations were performed at the EOM-CCSD/d-aug-cc-pVTZ level. Comparison with selected theoretical^23,37–39^ and experimental results^24,40–42^ from the literature shows an excellent agreement. MS-CASPT2,^23^ multistate CASPT2; TD-DFT/HTCH­(AC),^37^ time-dependent density-functional using the asymptotically corrected HCTH functional; GMS-SU-CCSD,^38^ general-model-space state-universal CCSD; exFCI,^39^ extrapolated full configuration interaction from a selected configuration interaction calculation. Basis sets in which each calculation was performed are also shown.

For the stabilization procedure, we systematically varied the diffuseness of the basis set by multiplying the exponents of all augmented functions—both the “d” and “aug” functions of the d-aug-cc-pVTZ basis set—by the stabilization parameter α. Since the doubly augmented basis set family includes additional diffuse functions,^34^ we used values of α up to 25 to ensure a truly compact basis set. At α = 25, the exponent of the most diffuse functions becomes comparable to that of the most diffuse function in the corresponding cc-pVTZ basis set,^43^ effectively eliminating the diffuse character and ensuring a compact basis set.

To evaluate the character of a given electronically excited state using the stabilization method, its vertical excitation energy curve in the stabilization plot must be followed diabatically from the calculation with the default basis set (α = 1) onward. In this context, avoided crossings observed in the plot are interpreted as true crossings between states. Diabatic tracking is achieved by following curves with similar slopes through the avoided crossings and by monitoring the character of the MOs involved in the dominant electronic configurations. It is important to note here that the character of the MO is used only to track the diabatic character of the curves, not to assign the nature of the electronically excited state. An illustrative example of this procedure is shown in Figure 1. In this case, an avoided crossing is observed between states S_1_ and S_2_ at α_1_. From a diabatic perspective, state S_1_ evolves into state S_2_ after the crossing, and vice versa. Based on this point of view, S_1_ would be characterized as Rydberg or mixed valence–Rydberg with a strong Rydberg component, while S_2_ would be assigned as valence. Notably, both of these states can be followed diabatically across the entire range of α values considered. In contrast, state S_3_ cannot be diabatically tracked for all values of α. It exhibits Rydberg character up to α_2_, but beyond this point it acquires a valence character due to an interaction with a higher-lying valence state not included in the computed manifold, that is, if one were to compute a larger number of electronically excited states, state S_3_ would have an avoided crossing with a higher-lying valence state at α_2_. Although S_3_ can be identified as Rydberg based on its behavior below α_2_, it cannot be consistently followed diabatically throughout the full α range.

**Figure 1:**
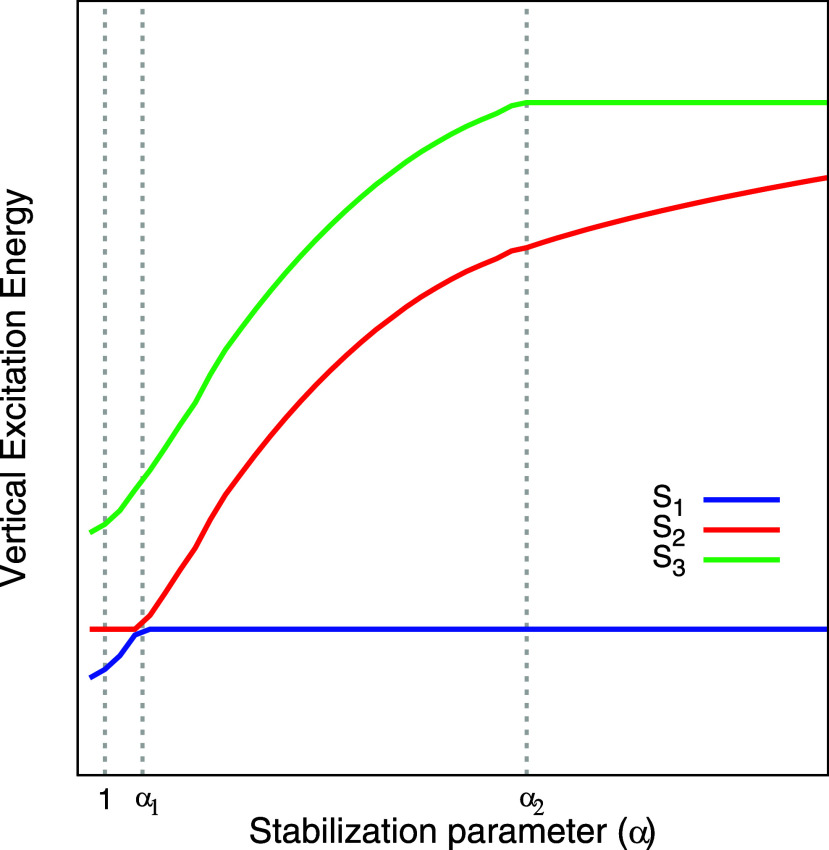
Illustrative example of stabilization plots. See text for discussion.

To investigate Rydbergization in the low-lying excited states of water, the stabilization procedure was applied along both the symmetric and asymmetric dissociation pathways. To this end, two sets of geometries were generated: one in which both O–H bonds are stretched simultaneously (symmetric dissociation), and another in which only one O–H bond is stretched while the other remains fixed at the ground-state equilibrium length (asymmetric dissociation). In both cases, the H–O–H bond angle was kept constant at its ground-state value. Although the bond angle is expected to vary during dissociation, we kept it fixed to reduce the computational effort and to attain an interpretation of the results. At each geometry, the stabilization procedure was carried out by varying the diffuseness of the basis set through the stabilization parameter α. We then computed for selected states the energy difference between the results for the most compact basis set used (α = 25) and the default basis set (α = 1). Hereafter, this energy difference is denoted as Δ*E*. To compute this energy difference for a given state, the associated stabilization curve is followed diabatically, as discussed in the previous paragraph. For instance, suppose that we are to compute the energy difference of state 2^1^ *B*_2_ at a given geometry. Suppose also that the previous state, 1^1^ *B*_2_, has a Rydberg nature, such that as α increases (more compact basis set), there is an avoided crossing between these states. In this case, diabatically, at α = 25, state 1^1^ *B*_2_ corresponds to state 2^1^ *B*_2_ at α = 1. Thus, the energy difference would be computed as Δ*E* (2^1^ *B*_2_) = *E*(1^1^ *B*_2_; α = 25) – *E*(2^1^ *B*_2_;α = 1). This example is actually what happens with state 2^1^ *B*_2_ for water at the equilibrium geometry, as will be shown in the next section, and is analogous to states S_1_ and S_2_ of Figure 1. Generically, the energy difference of a given state *N* ^1^Γ, corresponding to the *N*th state belonging to symmetry Γ, calculated with the default basis set (α = 1), may be computed as

1 $$\Delta E\left(N^{1}\Gamma \right)=E\left(M^{1}\Gamma ;\alpha =25\right)-E\left(N^{1}\Gamma ;\alpha =1\right).$$

In eq 1, *M* ^1^Γ is the corresponding diabatic state to *N* ^1^Γ computed at α = 25. If, in the stabilization plots, *N* ^1^Γ does not show any avoided crossings, *N* = *M*. Conversely, if there are any avoided crossings in the stabilization graphs, *N* ≠ *M*.

In an ideal scenario, valence states, being insensitive to the basis set diffuseness, would yield Δ*E* = 0, while Rydberg states would show Δ*E* > 0. In practice, valence states exhibit small but nonzero Δ*E* values. This is associated with the inherent approximations and numerical precision of quantum chemical calculations. Based on the results presented here and previous calculations,^27^ we propose using a threshold of Δ*E* = 0.1 eV to assign valence character; that is, states with Δ*E* < 0.1 eV are classified as valence states. Mixed states with a significant valence component typically have Δ*E* values close to, but not below, this threshold. For these kinds of mixed states, we proposed a threshold of 1.0 eV. Rydberg states and mixed states with a dominant Rydberg character exhibit significantly larger Δ*E* values. In order to illustrate this point, Δ*E* values and assignments of some excited states are presented in Table 2. Although these thresholds introduce a degree of arbitrariness in the SM, we note here that this criterion is molecule-independent, thus being more general than assignments based on electronic spatial radial distributions. Nonetheless, by plotting Δ*E* against bond length, the Rydbergization process can be effectively tracked using the stabilization method: if, for a given state, Δ*E* decreases to values close to zero as the dissociation progresses, Rydbergization occurs. On the other hand, if Δ*E* retains a considerable value through dissociation, the nature of that electronically excited state remains the same as the nuclei relax.

**Table 2:** Δ*E* (in eV) Values and Assignments of Some Electronically Excited States for Water, CCl_4_, HCOOH, and C_7_H_7_Cl^a^

water			CCl_4_			HCOOH			C_7_H_7_Cl		
state	Δ*E*	assignment	state	Δ*E*	assignment	state	Δ*E*	assignment	state	Δ*E*	assignment
2^1^ *A*_1_	0.470	MV	1^1^T_1_	0.055	V	1^1^ *A*″	0.061	V	2^1^ *A*′	0.050	V
3^1^ *A*_1_	7.025	R/MR	1^1^T_2_	0.053	V	2^1^ *A*′	0.435	MV	3^1^ *A*′	0.072	V
1^1^ *A*_2_	0.595	MV	1^1^E	0.075	V	3^1^ *A*′	0.686	MV	1^1^ *A*″	0.314	MV
2^1^ *A*_2_	3.309	R/MR	1^1^ *A*_2_	0.076	V	2^1^ *A*″	0.336	MV	2^1^ *A*″	0.685	MV
1^1^ *B*_1_	0.289	MV	2^1^T_1_	0.081	V	4^1^ *A*′	0.414	MV	3^1^ *A*″	0.404	MV
2^1^ *B*_1_	8.190	R/MR	2^1^T_2_	0.081	V	5^1^ *A*′	1.942	R/MR	4^1^ *A*″	0.662	MV
3^1^ *B*_1_	4.197	R/MR	2^1^E	0.086	V	3^1^ *A*″	2.808	R/MR	5^1^ *A*″	0.697	MV
1^1^ *B*_2_	10.168	R/MR	3^1^T_1_	0.064	V	4^1^ *A*″	0.652	MV	4^1^ *A*′	0.138	MV
2^1^ *B*_2_	0.689	MV	3^1^E	0.080	V	5^1^ *A*″	0.088	V	6^1^ *A*″	0.778	MV

a Information regarding the former three molecules was taken from ref 27. The underlying electronic structure calculation for each molecule was: water, EOM-CCSD/d-aug-cc-pVTZ; CCl_4_: TD-DFT/PBE0/aug-cc-pVDZ; HCOOH: TD-DFT/CAM-B3LYP/aug-cc-pVDZ; C_7_H_7_Cl: TD-DFT/B3LYP/aug-cc-pVDZ. All calculations were performed at the optimized ground-state geometry. V: valence, MV: mixed state with a strong valence component, R/MR: Rydberg or mixed state with a strong Rydberg component.

## Results and Discussion

### Equilibrium Geometry

Before proceeding with the Rydbergization analysis, we briefly comment on the level of theory employed in this study. Table 1 presents the vertical excitation energies obtained at the EOM-CCSD/d-aug-cc-pVTZ level, alongside selected experimental data^24,40–42^ and theoretical results^23,37–39^ from the literature. The results show excellent agreement with both experimental measurements and high-level theoretical calculations, even those with significantly higher computational cost. Some discrepancies are observed in the comparison with older experimental data,^41,42^ particularly for the higher-lying states. These differences may be a consequence of uncertainties in earlier assignments of absorption bands, made in the absence of high-level quantum chemical methods. When comparing to theoretical results,^23,37–39^ the present EOM-CCSD/d-aug-cc-pVTZ calculations present a overall good agreement, with the most notable discrepancies appearing in comparison with the higher-lying states reported by Li and Paldus.^38^ Our results are in better agreement with experimental data, likely due to the absence of extra diffuse functions in the basis sets used in ref 38. Cai et al.^37^ have emphasized the importance of including such functions to accurately describe the higher-lying electronically excited states of water.

Given that the stabilization procedure requires multiple calculations at each geometry, the use of the EOM-CCSD method with the d-aug-cc-pVTZ basis set offers a favorable balance between computational cost and accuracy. As shown in Table 1, this level of theory provides a reliable description of electronically excited states, making it suitable for the present study.

Additionally, the stabilization graphs for the lower electronically excited states of water at the equilibrium geometry, grouped by symmetry, are shown in Figure 2. Although no purely valence states are observed, states 2^1^ *A*_1_, 1^1^ *A*_2_, and 1^1^ *B*_1_ exhibit a strong valence character. These states show only a small energy variation ranging from 0.2 to 0.5 eV—between α = 1 and α = 25, indicating a small Rydberg character in their wave functions. Accordingly, they are classified as mixed valence-Rydberg states. Note that these states do not present any avoided crossings with other states in the stabilization graphs shown in Figure 2. Thus, to compute the respective energy differences, we have the case where *N* = *M* in eq 1.

**Figure 2:**
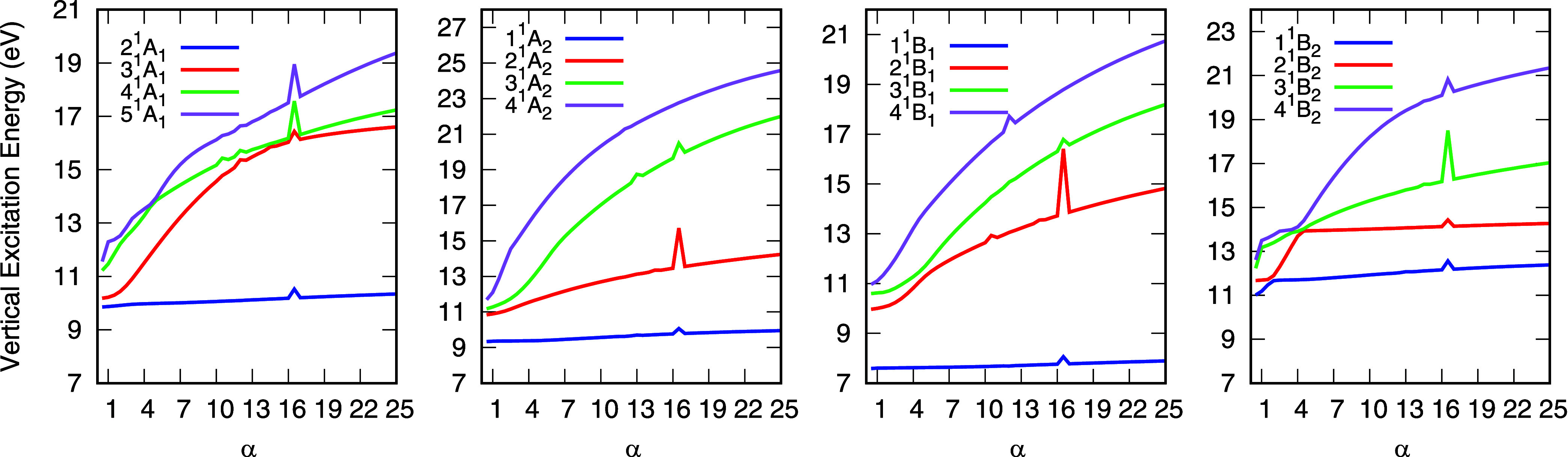
Stabilization graph for the lowest lying electronically excited states of water obtained through an EOM-CCSD/d-aug-cc-pVTZ calculation at the equilibrium geometry. Exponents from both “d” and “aug” functions were scaled by the stabilization parameter α.

For the *B*_2_ symmetry, two excited states exhibit significant valence character (Figure 2). One is the state labeled 2^1^ *B*_2_ at α = 1, which becomes 1^1^ *B*_2_ as α increases. This is the example discussed in the previous section, with *N* = 2 and *M* = 1 in eq 1. The other valence-like state has a higher energy and, for α > 4, is identified as 2^1^ *B*_2_ in Figure 2. The corresponding state calculated with the default basis set (α = 1) lies above the energy range of the states analyzed here. The remaining excited states shown in Figure 2 are predominantly Rydberg in character, as evidenced by the pronounced increase in their energies when the basis set becomes more compact. Some numerical instabilities can also be seen, which gives rise to peaks in the stabilization plots as a consequence of linear dependencies in the basis set for selected values of α. Nevertheless, the assignments at the equilibrium and extended geometries are unaffected by these small numerical instabilities.^27^

The assignments made with the SM are consistent with those reported in the literature.^23,24,37–40^ More specifically, Rubio et al.^23^ conducted a thorough analysis of the nature of the electronically excited states of water. The states characterized here as mixed valence-Rydberg correspond to those with the lowest electronic radial spacial distribution reported in their work,^23^ providing further evidence that these states, at the equilibrium geometry, possess a mixed valence-Rydberg character. Since our primary focus is to investigate the Rydbergization process, we do not further explore their detailed characterization at the equilibrium geometry. For a more in-depth discussion regarding each state, the reader is referred to the literature.^23^

### Symmetric Dissociation

Figure 3 presents the PECs along the symmetric O–H bond dissociation pathway for the low-lying *A*_1_ singlet excited states of water, accompanied by selected stabilization plots. At compressed bond lengths, the 2^1^ *A*_1_ and 3^1^ *A*_1_ states are nearly degenerated, leading to strong configuration mixing. As the bonds elongate, the PECs of these states gradually separate, and the degree of mixing lowers, consistent with the findings of Rubio et al.^23^ Remarkably, the stabilization method can distinguish the character of these two states even at short bond lengths. In both the PECs and the stabilization plots, increasing either the bond lengths or the stabilization parameter, respectively, leads to the separation of the states. A similar behavior was observed in formic acid, where varying the stabilization parameter enabled the separation of a valence-like state from nearby Rydberg states, reflecting the trends seen in PECs.^27^

**Figure 3:**
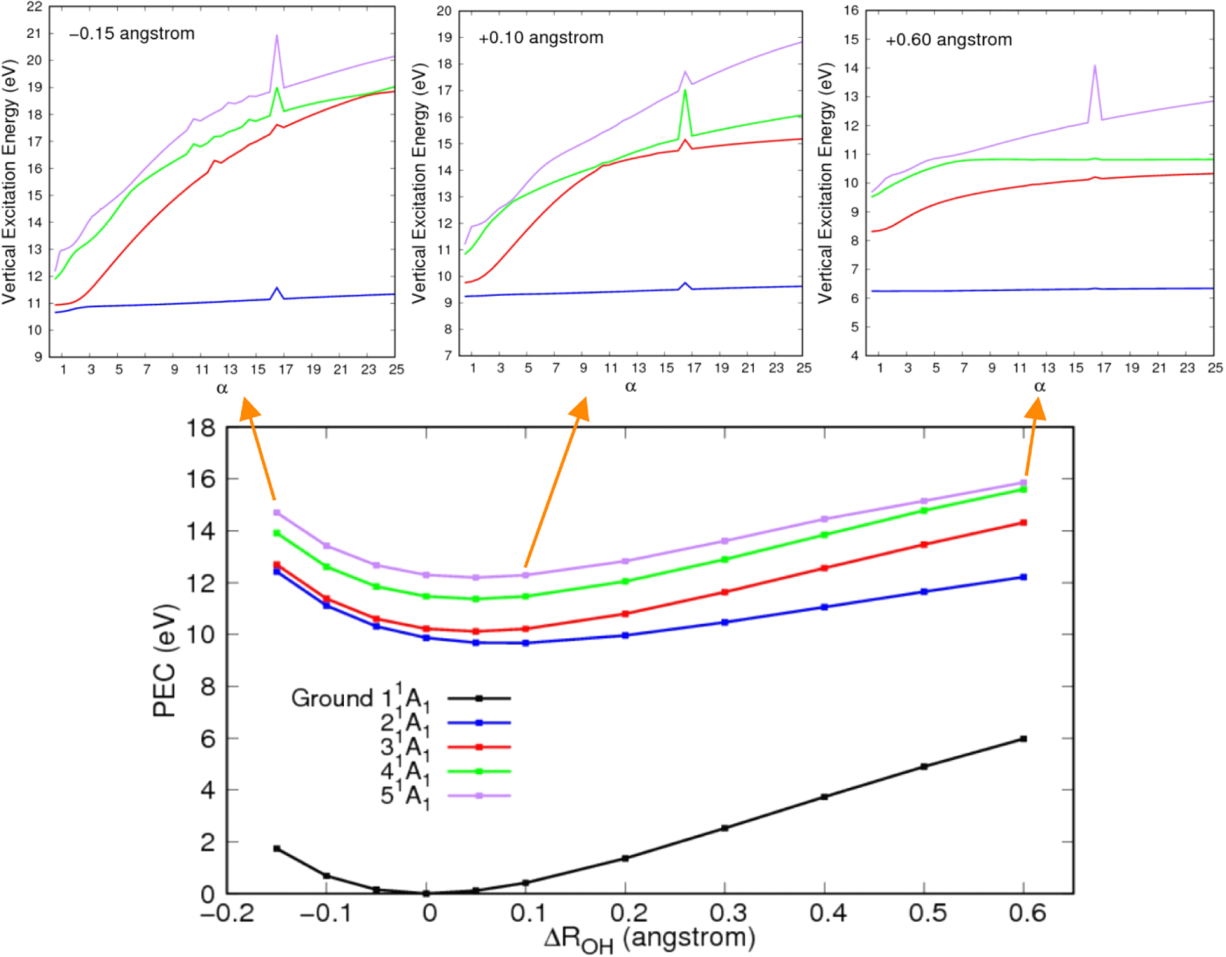
Potential energy curves (PECs) and selected stabilization graphs (upper panels) for the four lowest electronically excited states of *A*_1_ symmetry. As the bond stretches, the 1^1^ *A*_1_ state progressively acquires greater valence character (lower slope in the stabilization curves), indicating that the Rydbergization process occurs through this dissociation pathway.

Since states 4^1^ *A*_1_ and 5^1^ *A*_1_ possess a strong Rydberg character, following them diabatically in the stabilization graphs would require computing a larger number of excited states. The behavior of these states is analogous to that of state S_3_ shown in Figure 1. Therefore, values of Δ*E* were only computed for states that can be reliably tracked diabatically across all geometries considered. Evidently, if a state cannot be diabatically tracked, it is because it retains a Rydberg character throughout dissociation, and no Rydbergization occurs. In the case of *A*_1_ symmetry states, the analysis is restricted to the first and second electronically excited states, i.e., 2^1^ *A*_1_ and 3^1^ *A*_1_. As shown in the stabilization graphs in Figure 3, the overall slope of state 2^1^ *A*_1_ decreases with increasing O–H bond length, a behavior consistent with the expected Rydbergization trend. This trend becomes more evident when examining the energy difference between the state calculated with α = 1 (default basis set) and α = 25 (highest degree of compactness) as a function of bond length variation (Figure 4). The decreasing energy difference with increasing bond length shows that the valence character of the 2^1^ *A*_1_ state becomes more prominent as the bonds stretch. In contrast, the energy difference for the 3^1^ *A*_1_ state remains large across all geometries, suggesting that this excited state retains its Rydberg character throughout. These observations are in excellent agreement with the findings of Rubio et al.,^23^ further confirming the Rydbergization behavior of state 2^1^ *A*_1_ following the symmetric dissociation path. Furthermore, since there is an interaction between the first two electronically excited states as the O–H bond lengths decrease in value (Figure 3), the Rydbergization of 2^1^ *A*_1_ constitutes a case of MO-or-state Rydbergization, as pointed out by Rubio et al.^23^

**Figure 4:**
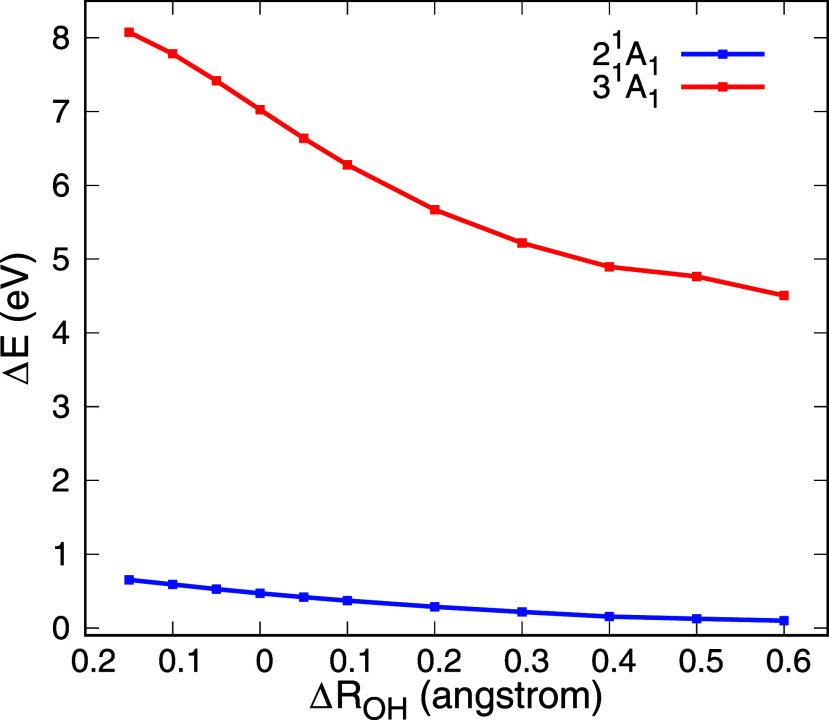
Difference in energy (Δ*E*) of each state calculated between α = 25 and α = 1 in the stabilization graphs as a function of the O–H bond length (Δ*R*_OH_). State 2^1^ *A*_1_ undergoes Rydbergization, while state 3^1^ *A*_1_ retains the Rydberg character for all geometries.

The same procedure used to analyze the states of *A*_1_ symmetry was applied to the low-lying electronically excited states of water with *A*_2_, *B*_1_, and *B*_2_ symmetries. The corresponding PECs and energy differences are presented in Figure 5. For the *A*_2_ and *B*_1_ symmetries, our results are in excellent agreement with those reported by Rubio et al.^23^ and the experimental assigment of Mota et al.^24^ In both cases, the first electronically excited state undergoes the Rydbergization process, while the higher-lying states retain a Rydberg character across all bond distances. The PECs for the 1^1^ *A*_2_ and 1^1^ *B*_1_ states show no signs of interaction with higher Rydberg states, indicating that Rydbergization occurs primarily at the MO level, consistent with the interpretation of Rubio et al.^23^ The strong overall agreement with previous work supports the reliability and effectiveness of the stabilization method employed in this study for describing Rydbergization in the electronically excited states of water. Furthermore, an avoided crossing is seen in the PECs of states 2^1^ *B*_1_ and 3^1^ *B*_1_ at bond extensions around 0.4 Å. This is reflected in the calculated energy differences between these states, which exhibit abrupt changes near the crossing point. Diabatically, state 2^1^ *B*_1_ evolves into state 3^1^ *B*_1_ after the crossing, while state 3^1^ *B*_1_ becomes 2^1^ *B*_1_. The same behavior was reported by Rubio et al.^23^

**Figure 5:**
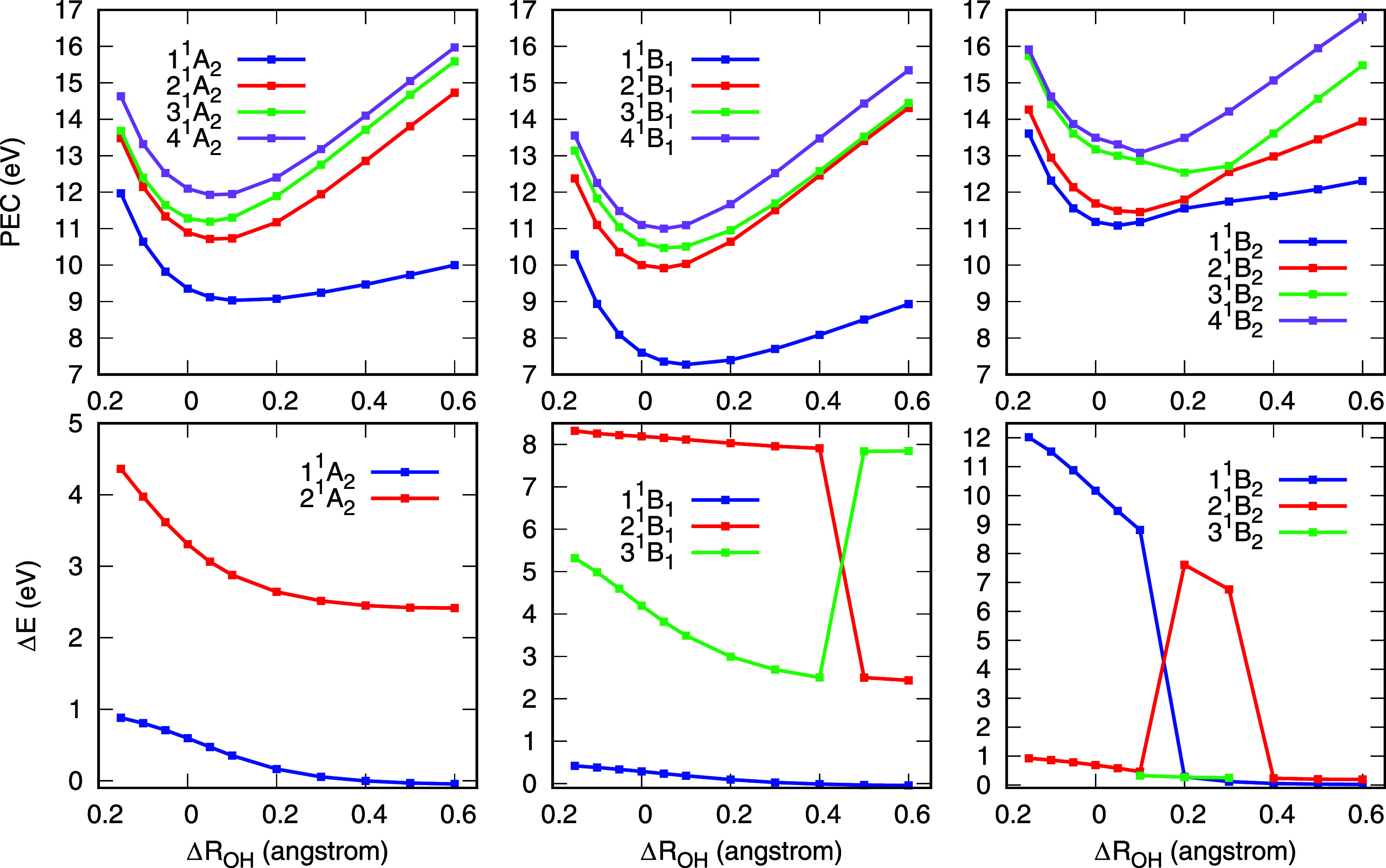
Potential energy curves (PEC, upper panels) and difference in energy (Δ*E*, lower panels) of each state calculated between α = 25 and α = 1 in the stabilization graphs as a function of the symmetric stretching of the O–H bond length (Δ*R*_OH_). Results for states of *A*_2_, *B*_1_, and *B*_2_ symmetries are presented in the first, second, and third columns, respectively. See text for discussion.

The PEC and energy difference plot for the *B*_2_ symmetry are also presented in Figure 5. As the O-H bonds stretch, avoided crossings between excited states become evident in the PECs. From a diabatic perspective, valence-like states cross Rydberg states, leading to abrupt jumps in the energy difference curves (as was the case for the 2^1^ *B*_1_ and 3^1^ *B*_1_ states). As discussed in the previous section, water has two valence-like states in the *B*_2_ symmetry, the first is state 2^1^ *B*_2_ at the equilibrium geometry. As the O–H bonds stretch, an avoided crossing is seen in the PEC of this state and state 1^1^ *B*_2_ around 0.15 Å (Figure 5). From this point forward, state 2^1^ *B*_2_ evolves into state 1^1^ *B*_2_. If one follows the energy difference of this state diabatically, considering state 2^1^ *B*_2_ for Δ*R*_OH_ up to 0.1 Å and state 1^1^ *B*_2_ above this value, one can see that the Rydbergization process does occur, that is, Δ*E* decreases with increasing bond lengths. Since there is an interaction with the nearby Rydberg state, this constitutes a case of MO-or-state Rydbergization.

In the *B*_2_ symmetry, water has another higher-lying valence-like state at the equilibrium geometry (Figure 2). At this geometry with the default basis set, this state lies above the state computed here, as discussed previously. As a result, we are unable to track its stabilization diabatically at the equilibrium geometry and, consequently, cannot compute Δ*E* for this state at this geometry. However, as the bond stretches, this higher-lying valence-like state undergoes avoided crossings with the lower-lying Rydberg states (Figure 5). In the bond length range between 0.1 and 0.3 Å, this state becomes 3^1^ *B*_2_, while for bond extensions of 0.4 Å and beyond, it corresponds to 2^1^ *B*_2_. In these regions of the dissociation path, we are able to compute Δ*E*, as shown in Figure 5. Thus, Rydbergization also occurs for this second valence-like state through an MO-or-state process, driven by state interactions and avoided crossings between different excited states—distinct from the purely MO Rydbergization seen in the *A*_2_ and *B*_1_ symmetries.^23^ To the best of our knowledge, this is the first time that the Rydbergization process has been explicitly characterized for water’s electronically excited states of *B*_2_ symmetry following the symmetric dissociation path.

### Asymmetric Dissociation

In the previous section, we demonstrated that the stabilization method effectively captures the Rydbergization phenomenon in the low-lying excited states of water along the symmetric dissociation pathway. We now extend this analysis to the asymmetric dissociation pathway to investigate whether a similar behavior is observed. To the best of our knowledge, the Rydbergization of low-lying states along this dissociation route has only been previously explored in early SCF calculations by Horsley and Fink,^22^ who reported evidence of Rydbergization in the 2^1^ *A*_1_ state. In this work, we reexamine the Rydbergization process of this and other low-lying states using a state-of-the-art quantum chemical method.

In the asymmetric dissociation pathway, OH + H products are formed. To investigate Rydbergization along this pathway, one O–H bond was stretched while the other was held fixed at its ground-state equilibrium length. The bond angle was kept constant at the ground-state equilibrium geometry value. This dissociation path lowers the molecular symmetry from *C*_2*v*_ to *C*_*s*_. Under this reduced symmetry, states belonging to the *A*_1_ and *B*_2_ irreducible representations correlate with the *A*′ irreducible representation, whereas states of *A*_2_ and *B*_1_ symmetry correlate with the *A*″ symmetry. For each geometry considered, the stabilization procedure was performed for the first 6 low-lying states of each symmetry. Afterward, the energy difference of a given state calculated with α = 1 and α = 25 as a function of *R*_OH_ coordinate variation was monitored to probe Rydbergization.

The PECs and the energy differences are presented in Figure 6. For the *A*′ symmetry, it is observed that the PEC of the first electronically excited state, 2^1^ *A*′, is repulsive and undergoes Rydbergization as the bond dissociates. This is in good agreement with the early findings of Horsley and Fink.^22^ As the bond length decreases, this state approaches 3^1^ *A*′, which maintains a Rydberg character across all geometries considered. These two states correspond to the 2^1^ *A*_1_ and 3^1^ *A*_1_ states at the equilibrium geometry, respectively. Thus, for these states, the same behavior observed along the symmetric dissociation pathway is also found here.

**Figure 6:**
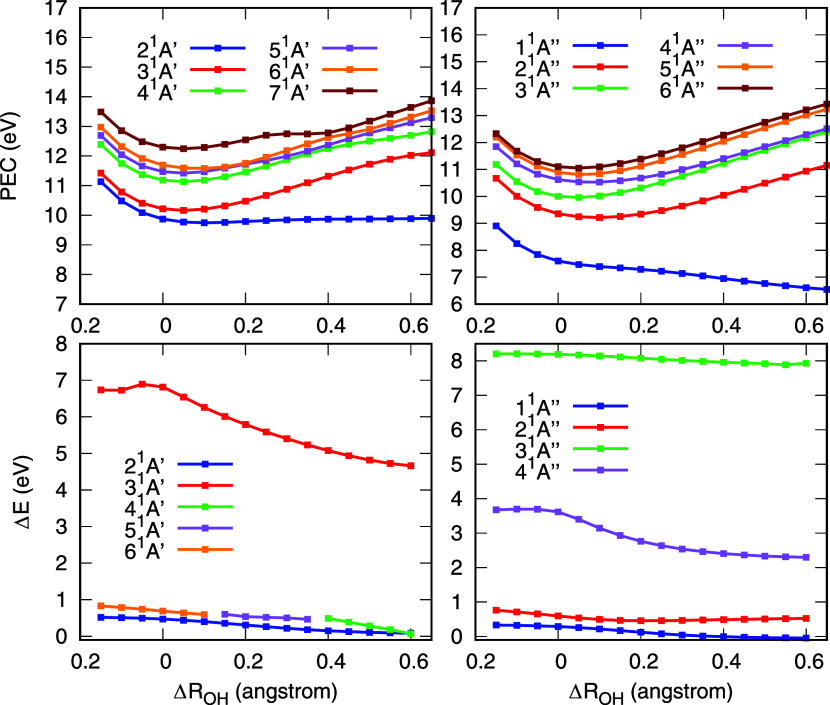
Potential energy curves (PEC, upper panels) and difference in energy (Δ*E*, lower panels) of each state calculated between α = 25 and α = 1 in the stabilization graphs as a function of the symmetric stretching of the O–H bond length (Δ*R*_OH_). Results for states of *A*′ and *A*″ symmetries are presented in the left and right columns, respectively. See text for discussion.

Beyond these states, another valence-like *A*′ state also undergoes Rydbergization as the bond stretches (Figure 6). This state corresponds to 6^1^ *A*′ for bond extensions between −0.15 and + 0.10 Å, 5^1^ *A*′ between + 0.15 and + 0.35 Å, and 4^1^ *A*′ for extensions greater than or equal to + 0.40 Å. The respective avoided crossings are visible in the PECs. This second valence-like state corresponds to the 2^1^ *B*_2_ state at the equilibrium geometry. Therefore, as in the symmetric dissociation pathway, both the 2^1^ *A*_1_ and 2^1^ *B*_2_ states undergo MO-or-state Rydbergization along the asymmetric dissociation pathway.

The first electronically excited state of *B*_1_ symmetry (1^1^ *B*_1_), corresponding to state 1^1^ *A*″ in Figure 6, exhibits a repulsive PEC. This behavior is consistent with its known dissociation into OH + H products,^25,26^ and is therefore expected. Furthermore, the state undergoes Rydbergization without interacting with any other state of the same symmetry. Thus, the same MO Rydbergization observed for this state along the symmetric dissociation pathway is also present when only one O-H bond is stretched. These conclusions are in good agreement with the experimental evidence from Chergui and Schwentner.^21^

In contrast, the behavior of the 2^1^ *A*″ state is notably different. This state corresponds to the 1^1^ *A*_2_ state at the equilibrium geometry. Along the symmetric dissociation pathway (Figure 5), this state undergoes Rydbergization, as discussed in the previous section. However, along the asymmetric dissociation path, the calculated energy difference remains relatively unchanged, as shown in Figure 6. That is, the overall slope of the curves associated with this state in the stabilization graphs remains approximately the same as the O–H bond stretches. This indicates that the valence component of the wave function does not increase with bond length. Thus, Rydbergization does not take place. Moreover, the potential energy curve of this state differs significantly depending on the dissociation mode. Along the asymmetric pathway, the PEC is bound, while in the symmetric pathway, it features a shallower well, facilitating dissociation. Thus, the 1^1^ *A*_2_ state exhibits markedly different behavior depending on the dissociation pathway: in the symmetric case, the PEC facilitates dissociation and Rydbergization occurs; in the asymmetric case, the PEC is bound and no Rydbergization takes place. The remaining states analyzed here retain a Rydberg character for all geometries considered.

## Conclusions

In this work, we investigated the Rydbergization process of the low-lying electronically excited states of water using the stabilization method. The underlying electronic structure calculations were performed with EOM-CCSD and the d-aug-cc-pVTZ basis set. Both ”d” and ”aug” functions were scaled by the stabilization parameter. First, along the symmetric dissociation pathway, we confirmed that the stabilization method is capable of reproducing the findings of Rubio et al.^23^ for states of 2^1^ *A*_1_, 1^1^ *A*_2_, and 1^1^ *B*_1_ symmetry. While states 1^1^ *A*_2_ and 1^1^ *B*_1_ undergo Rydbergization at the MO level, state 2^1^ *A*_1_ interacts with nearby Rydberg states during dissociation. Additionally, novel results were presented for states of *B*_2_ symmetry. In this case, two valence-like states at the equilibrium geometry undergo MO-or-state Rydbergization, characterized by multiple avoided crossings with other Rydberg states. These latter results for the *B*_2_ symmetry states provide new data regarding the low-lying singlet states of water.

The Rydbergization process was also examined along the asymmetric dissociation pathway, providing new results obtained with a state-of-the-art electronic structure method. The most striking difference lies in the behavior of the 1^1^ *A*_2_ state: whereas it undergoes Rydbergization along the symmetric pathway, this transformation does not occur when only one O–H bond is stretched. This highlights that dissociation dynamics can be highly pathway-dependent and that conclusions drawn from one geometric distortion cannot be uncritically generalized to others. Thus, a comprehensive understanding of excited-state behavior requires careful consideration of multiple dissociation coordinates. Thus, in this contribution, we have proved that the stabilization method is capable of describing subtle processes, such as the Rydbergization phenomenon, and provided new data regarding the Rydbergization of the low-lying electronically excited states of water.
